# Barrier Height, Ideality Factor and Role of Inhomogeneities at the AlGaN/GaN Interface in GaN Nanowire Wrap-Gate Transistor

**DOI:** 10.3390/nano13243159

**Published:** 2023-12-17

**Authors:** Siva Pratap Reddy Mallem, Peddathimula Puneetha, Yeojin Choi, Seung Mun Baek, Dong-Yeon Lee, Ki-Sik Im, Sung Jin An

**Affiliations:** 1 Advanced Material Research Center, Kumoh National Institute of Technology, Gumi 39177, Republic of Korea; drmspreddy@kumoh.ac.kr; 2 Department of Robotics and Intelligent Machine Engineering, College of Mechanical and IT Engineering, Yeungnam University, Gyeongsan 38541, Republic of Korea; puneethaphd@gmail.com (P.P.); dylee@ynu.ac.kr (D.-Y.L.); 3 Department of Materials Science and Engineering, Kumoh National Institute of Technology, Gumi 39177, Republic of Korea; dota23@kumoh.ac.kr (Y.C.); monndal980@kumoh.ac.kr (S.M.B.); 4 Department of Green Semiconductor System, Daegu Campus, Korea Polytechnics, Daegu 41765, Republic of Korea

**Keywords:** wrap-gate transistor, nanowire, GaN, barrier height, inhomogeneities

## Abstract

It is essential to understand the barrier height, ideality factor, and role of inhomogeneities at the metal/semiconductor interfaces in nanowires for the development of next generation nanoscale devices. Here, we investigate the drain current (*I*_ds_)–gate voltage (*V*_gs_) characteristics of GaN nanowire wrap-gate transistors (WGTs) for various gate potentials in the wide temperature range of 130–310 K. An anomalous reduction in the experimental barrier height and rise in the ideality factor with reducing the temperature have been perceived. It is noteworthy that the variations in barrier height and ideality factor are attributed to the spatial barrier inhomogeneities at the AlGaN/GaN interface in the GaN nanowire WGTs by assuming a double Gaussian distribution of barrier heights at 310–190 K (distribution 1) and 190–130 K (distribution 2). The standard deviation for distribution 2 is lower than that of distribution 1, which suggests that distribution 2 reflects more homogeneity at the AlGaN/GaN interface in the transistor’s source/drain regions than distribution 1.

## 1. Introduction

Nano-based field-effect transistors (FET) are involved with replacing existing conventional technology and have surged to be one of the potential solutions towards continuous complementary metal-oxide-semiconductor (CMOS) scaling. The semiconductor-based nanowires have been significantly explored because of their most promising applications for next-generation high-quality optoelectronic/electronic devices [1,2,3]. The scaling of numerous transistor types is based on geometries as in Fin-FET, omega gate, tri-gate, and wrap-gate (WG) or gate-all-around (GAA) devices [4,5,6,7,8,9,10,11]. WG-based devices contribute particularly remarkable performance advantages over other geometries because of their extreme electrostatic controls. During recent years, WG-based devices fabricated by bottom-up techniques have been intensively studied as the fundamental building block for nano-electronic devices and circuit technologies. The top-down fabrication of nanowire WG transistors (WGTs) based on a sacrificial layer has many advantages in contrast to a bottom-up approach: reduced device size, large-scale feasibility with high yield, and an orderly alignment of parallel nanowires. It has already been reported that GaN-based nanowires have made significant progress. GaN-based devices have attractive features for great device performance such as high-speed, high-power, high-frequency, and high-temperature operations. A GaN nanowire-based device can control its normally off state with a high *I*_on_/*I*_off_ ratio, a low gate leakage current, and a high conductance. For this paper, we fabricated a device using the top-down process and investigated the electrical transport properties in a GaN nanowire WGT on a GaN-on-insulator (GaNOI) substrate.

Knowledge of nanowire device operation at cryogenic temperatures is of special interest because, at room temperature, nanowire devices do not yield broad information about their carrier transport characteristics. The current–voltage (*I–V*) properties of nanowires as a function of temperature allows the identification of various current flow mechanisms and additional significant electrical transport across the metal/semiconductor interface (i.e., source/drain regions) [12,13]. A good understanding of barrier heights, ideality factor, and the role of inhomogeneities at the metal/semiconductor interface in nanowires led to the concept of the thermionic emission (TE) mechanism by assuming the coexistence of a double Gaussian distribution.

The metal/semiconductor interface is characterized by a certain work function. The fact it is Ohmic or Schottky depends on the boundary conditions at the interface which depends on a few variables, including the semiconductor doping concertation near the interface. High doping allows the tuning of the carriers across the barriers, reducing the contact resistance. Usually, after the preparation of Ohmic contacts for the fabrication of FETs in their source and drain regions, certain semiconductor contacts appear to have Ohmic behavior, but in truth, they are Schottky in nature. In experiments, a FET with two identical source and drain regions can be seen to have back-to-back Schottky contacts.

Here, we have studied the electrical transport mechanism in the GaN nanowire WGT as a function of temperature with variable gate bias. The temperature dependence of the barrier height (Φ_b0_) and the ideality factor (*η*) values are found at the interface of AlGaN/GaN in the source/drain regions of the GaN nanowire WGT. Finally, we showed that the two linear regions in the variation of Φ_b0_ versus 1/2 kT could be explained with the help of a double Gaussian distribution.

## 2. Materials and Methods

For the GaN nanowire WGT architecture, we used a GaNOI substrate based on Smart Cut^TM^ technology from the SOITEC Company by the dual-wafer transfer method [7]. A 4-inch diameter GaNOI substrate consists of 150 nm thick GaN film and 800 nm thick silicon-dioxide (SiO_2_) on a 0.65 mm thick sapphire wafer. Initially, the ‹11-20› phase was aligned by electron beam (e-beam) lithography on the GaNOI substrate with the help of poly(methyl methacrylate) (PMMA) resist. Using an inductively coupled plasma (ICP) dry etching process, the GaN film was selectively etched, and after this, the aligned film was dipped in 5% of tetramethylammonium hydroxide (i.e., TMAH was purchased from Transene company, Inc., Danvers, MA, USA) etchant solution at 90 °C for 10 min. The TMAH etchant solution entirely etches in the sideways direction instead of perpendicular *c*-plane (0001) direction. This etchant solution reduced the GaN film width along the ‹11-00› phase, giving 83 nm heights with similar triangular-shaped sidewall ‹11-01› phases. Next, the film was treated with a buffer-oxide etchant (BOE) solution to effectively remove the SiO_2_ layer under the structure of the GaN nanowires.

Next, selectively re-grow 50 nm and 20 nm thicknesses of undoped GaN and AlGaN films on the aligned GaN surface using a sophisticated metal-organic chemical vapor deposition (MOCVD) instrument. Here, a self-limiting re-growth in the ‹11-01› phase acted as an *r*-plane on the aligned GaN film. At this viewpoint, the surface of the *r*-plane consisted of nitrogen (N) atoms that were easily impelled by hydrogen (H) atoms in the MOCVD instrument and produced N-H bonds that limited growth and added stability in the plane direction. Hence, AlGaN/GaN films were not re-grown on the GaN nanowire but easily re-grown only on the source and drain regions. As a result of this procedure, the GaN nanowire’s area has not changed. In fact, the significance of re-grown AlGaN/GaN films on the source and drain regions decreases the series resistance through two-dimensional electron gas (2DEG) at the junction.

For WGT device fabrication, 10 nm and 20 nm thicknesses of gate-metal (TiN) and high-*k* gate oxide (Al_2_O_3_) were coated using plasma-enhanced atomic layer deposition (PE-ALD) method. The thicknesses of the coated materials are confirmed by the number of PE-ALD cycles, a deposition rate (growth per cycle) of ~0.1 nm/cycle was determined by an ellipsometry. Here, 100 and 200 PE-ALD cycles are relatively equal to ~10 nm and ~20 nm thickness of TiN and Al_2_O_3_ layers. Accordingly, metal layers (Ti/Al//Ni/Au) were coated as source and drain regions with an e-beam technique and followed by rapid thermal process at 850 °C for 30 s in N_2_ atmosphere. Finally, gate metal (Ni/Au) was coated as an outer contact for device measurements. The re-grown AlGaN film mobility (*μ*_d_, 1630 cm^2^/V·s) and concentration (*N*_S_, 9.75 × 10^12^ cm^−2^) were confirmed using Hall-effect examination (Nanometrics, HL5500PC, Kanata, ON, Canada). The device architecture was examined with a field-emission tunneling electron microscope (FE-TEM, 200 kV FE, JEM-2100F, Tokyo, Japan). The temperature-dependent *I*–*V* characteristics of the device were measured with a Keithley source unit (SCS-4200, Cleveland, OH, USA) connected to vacuum chamber (MST-6VC) with a low-temperature regulate system. The sensitivity of the temperature control system is ±1 K.

## 3. Results

**Figure 1:**
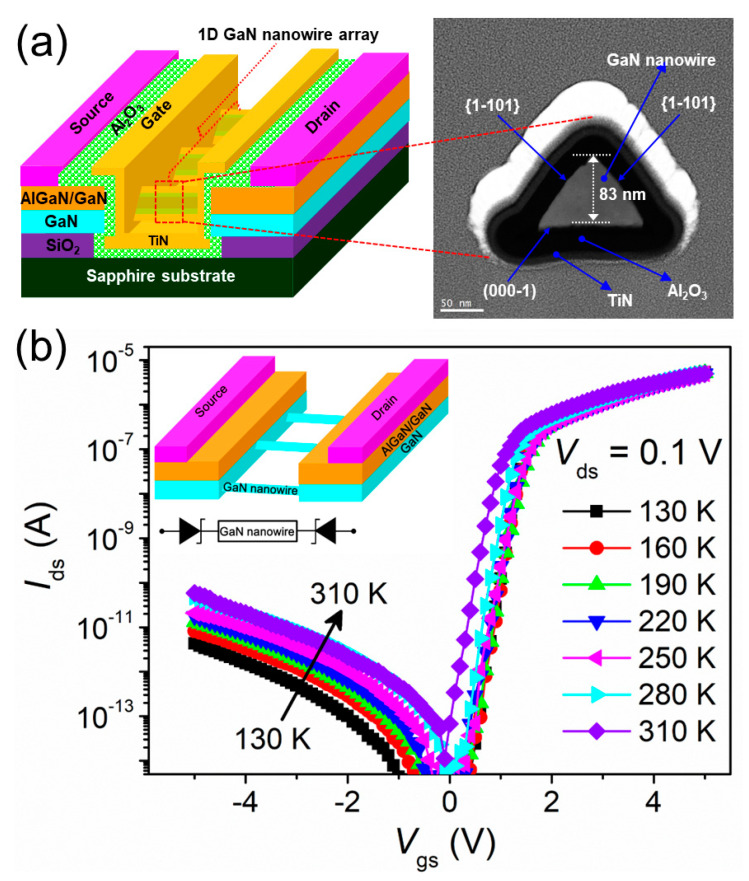
(**a**) Schematic device architecture of the fabricated GaN nanowire WGT with a high-resolution FE-TEM cross-section image of a triangular-shaped GaN nanowire. (**b**) Logarithmic plots of drain-current (*I*_ds_) versus gate-voltage (*V*_gs_) at *V*_ds_ = 0.1 V as a function of temperature, and inset figure shows back-to-back GaN nanowire WGT with a simplified circuit diagram.

Figure 1a (on the left side) illustrates the schematic architecture of the studied GaN nanowire WGT device. It has a 2 μm gate length consisting of 64 triangular-shaped one-dimensional nanowires, each having two similar ‹1-101› crystal facets. On the right side of Figure 1a, an FE-TEM (i.e., dark field mode) image clearly shows a triangular-shaped GaN nanowire core surrounded by gate-oxide and gate-metal. Figure 1b shows the drain current (*I*_ds_) versus gate voltage (*V*_gs_) curves of the AlGaN/GaN-based GaN nanowire WGT as a function of temperature ranging from 130 to 310 K in steps of 30 K at a drain voltage of *V*_ds_ = 0.1 V. The drain leakage current (*I*_ds_) clearly increases with temperature from 1.12 × 10^−13^ (at 130 K) to 2.15 × 10^−12^ A (at 310 K) at a *V*_gs_ of −2 V. The increase in drain leakage current may be due to the surface-related traps and temperature-assisted tunneling mechanisms [14,15]. The inset of Figure 1b shows a simplified diagram of the fabricated device with a back-to-back Schottky configuration composed of two (source/drain) contacts in series with GaN nanowires.

The current across the barrier mainly consists of three types of electron transport mechanisms: thermionic emission (TE), thermionic field emission (TFE), and field emission (FE). The dominant mode of carrier transport can be determined from the characteristics of the tunneling parameter *E*_00_; *E*_00_ « *kT* for TE, *E*_00_ ≈ *kT* for TFE, and *E*_00_ » *kT* for FE. *E*_00_ is evaluated from the doping concentration (*N*_d_) of the measured semiconductor as in [16]:

(1) $$E_{00}=\left(\frac{qh}{kT}\right)\sqrt{\frac{N_{d}}{m^{*}\varepsilon_{S}}}$$

where *h* is Planck’s constant, *q* is the charge, *T* is the absolute temperature, *k* is the Boltzmann constant, *ε*_S_ is the permittivity of the GaN (i.e., *ε*_S_ = 9.2*ε*_0_), m* is effective mass (m* = 0.3 *m*_0_ [17], where *m*_0_ is mass of electron), and the value of *N*_d_ in the present work is ~5 × 10^16^ cm^−3^. *E*_00_ is calculated to be about 8 meV from Equation (1), which is smaller than the value of kT at room temperature. This means that TE is the dominant carrier transport mechanism in the GaN nanowire at room temperature.

**Figure 2:**
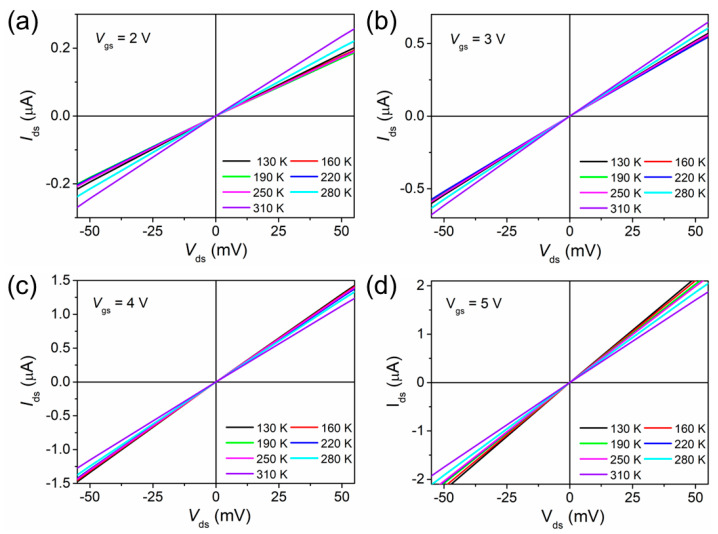
Drain-current (*I*_ds_) versus drain-voltage (*V*_ds_) plots of the GaN nanowire WGT as a function of temperature at gate biases of (**a**) 2 V, (**b**) 3 V, (**c**) 4 V, and (**d**) 5 V.

To investigate the effect of the GaN nanowire structure on the device characteristics at the AlGaN/GaN interface, we further analyzed the temperature dependence of effective barrier height (Φ_b0_) and ideality factor (*η*) at the interface for different gate potentials. A GaN nanowire WGT with two similar metal contacts at the AlGaN/GaN (source and drain) regions can be regarded as back-to-back Schottky diodes. In this device architecture, most of the voltage drop happens in the reverse-biased side [18,19]. The Φ_b0_ and *η* parameters are extracted by using the following relations [13]:

(2) $$I=I_{0}\exp\left(\frac{qV}{\eta kT}\right)\left[1-exp\left(-\frac{qV}{\eta kT}\right)\right]$$

where $I_{0}=A^{*}T^{2}{A\;exp\left(\frac{-q\Phi_{b0}}{kT}\right)}^{\;},\;$*V* is the gate bias, *A* is the contact area, and A* is the effective Richardson’s constant. The theoretical value of A* is ~35.8 Acm^−2^K^−2^ based on the effective mass of AlGaN [17] and is used for the calculation of Φ_b0_. Equation (1) is modified as follows:

(3) $$\;ln\left[\frac{I\exp\left(qV/kT\right)}{\exp(qV/kT)-1}\right]=lnI_{0}+\frac{qV}{\eta kT}$$

where *V* is the voltage drop across the junction, *V* = *V*_ds_ − *IR*, and here, *R* is the series resistance. The values of *I*_0_ at different gate potentials were obtained from the *I–V* measurements (Figure 2a–d) in the plot of ln[*I* exp(*qV*/*kT*/(exp(*qV*/*kT*) *−* 1)] versus *V* for the reverse bias at each temperature. The values of *η* and Φ_b0_ are extracted from the slope and y intercept using Equation (3).

**Figure 3:**
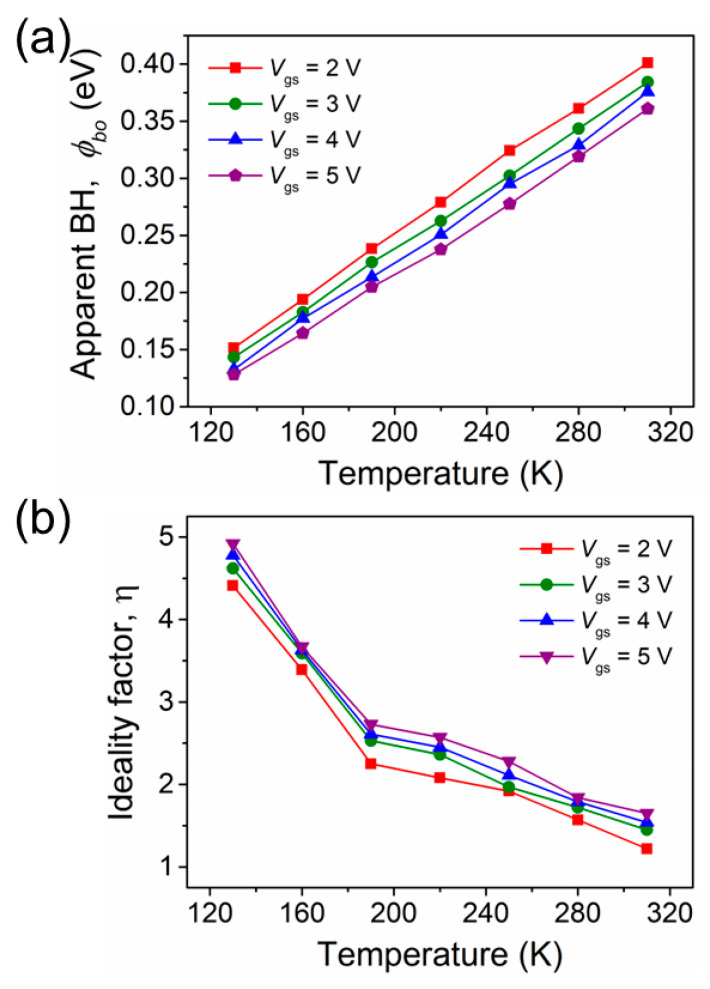
Variation in (**a**) Φ_b0_ and (**b**) η with temperature for different gate biases.

At temperature of 310 K, the values of Φ_b0_ (Figure 3a) and η (Figure 3b) for different gate voltages were, respectively, found to be 0.41 eV and 1.22 at 2 V, 0.39 eV and 1.45 at 3 V, 0.37 eV and 1.54 at 4 V, and 0.36 and 1.65 at 5 V. These values confirm that TE is the dominant current conduction mechanism in GaN nanowire-based devices at room temperature. It is clearly shown that Φ_b0_ (Figure 3a) increases and η (Figure 3b) decreases with increasing temperature at a range of gate voltages. At 130 K, the values of Φ_b0_ and η for various voltages were, respectively, found to be 0.15 eV and 4.4 at 2 V, 0.14 eV and 4.6 at 3 V, 0.12 eV and 4.8 at 4 V, and 0.11 eV and 4.9 at 5 V. It is worthwhile to note that these values of η are much better than found in previous studies of GaN-based nanowires and nano-rods [20,21,22,23]. This may be due to the low semiconductor doping concentration or influence of surface states at the interface compared to previous studies. These η values are higher than unity, however, indicating that TE is not the entire conduction mechanism. This typical signature may be the result of tunneling, interface states, electrical dipole formation, or barrier inhomogeneities [24,25]. Tunneling current is significant in nanoscale devices compared to bulk devices because the device size is comparable to or less than the zero bias depletion width [*W*_d_]. *W*_d_ can be expressed as follows [24,25]:

(4) $$W_{d}={\left(\frac{2\varepsilon_{S}\left(\Phi_{b0}-V_{n}\right)}{q^{2}N_{d}}\right)}^{\frac{1}{2}}$$

where *V_n_* = *kT*ln(*N_C_*/*N_d_*) is the position of the conduction band (*E*_C_) edge with respect to the Fermi level position (*E*_F_) in a GaN nanowire. *N*_C_ is the density of states in the conduction band minimum as given by *N_C_* = 2(2πm*kT/*h*^2^)^3/2^. Its values were ~9 × 10^17^ and 3.1 × 10^18^ cm^−3^ [24] at 130 K and 300 K. From Equation (4), the value of *W*_d_ varies in the range of 25–90 nm for all temperature barrier heights. These varied *W*_d_ values are comparable to a GaN nanowire height of ~83 nm, and hence, the tunneling current could be a major factor at the metal/AlGaN/GaN interface in GaN nanowires for all temperatures that drive the ideality factor above the unit value.

To further understand the nature of carrier transport in a GaN nanowire, we view an inhomogeneous metal contact at the AlGaN/GaN interface as a distribution of local high and low barrier height patches with nanoscale geometry. Here, electrical transport at low temperatures is dominated by low barrier height patches with a higher ideality factor as the carrier passes through the patches. At high temperatures, the carrier flows through high barrier patches causing the barrier height to increase and the ideality factor to decrease. Consequently, the barrier height at the AlGaN/GaN interface of the source/drain region is not constant but follows a Gaussian distribution due to the barrier inhomogeneities as in [26]:

(5) $$P\left(\Phi_{b0}\right)=\frac{1}{\sigma_{S}\sqrt{2\pi }}\exp\left[-\frac{{\left(\bar{\Phi_{b0}}-\Phi_{b0}\right)}^{2}}{2\sigma_{S}^{2}}\right]$$

Here, 1/*σ*_S_√2π is the normalized distribution constant, and $\bar{\Phi_{b0}}$ and Φ_b0_ are, respectively, the zero bias mean and the apparent barrier height. The standard deviation of the Gaussian distribution *σ*_S_ in the normalized distribution function *P*(Φ_b0_) represents the level of inhomogeneities at the interface of AlGaN/GaN in the GaN nanowire WGTs. $\bar{\Phi_{b0}}$ and Φ_b0_ (measured from semi-log *I*_ds_–*V*_gs_ data) are associated as in [27,28]:

(6) $$\Phi_{b0}=\bar{\Phi_{bo}}-\frac{\sigma_{S}^{2}}{2kT}$$

**Figure 4:**
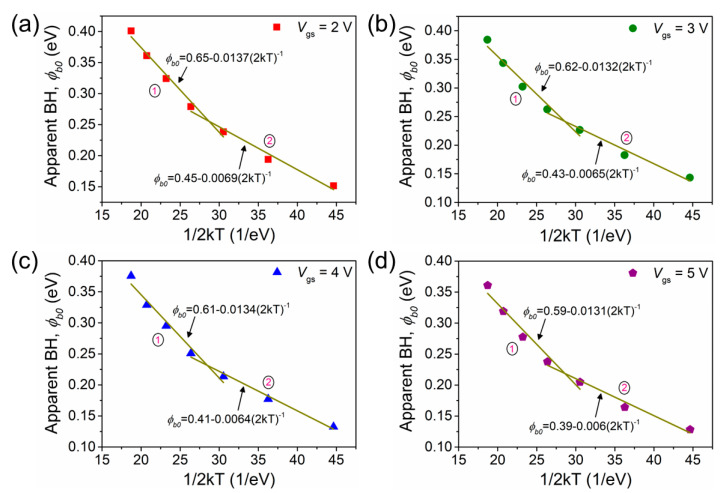
Apparent barrier height (Φ_b0_) as a function of 1/2 kT in the temperature range of 310–130 K for different gate biases (**a**) 2 V, (**b**) 3 V, (**c**) 4 V, and (**d**) 5 V. Solid straight lines show the least squares fit. Lower values of *σ*_S_ in the temperature range of 190–130 K (distribution 2) as compared to 310–190 K (distribution 1) indicate that the interface is more homogenous in the lower temperature region.

The above relation states that the effective Φ_b0_ is normally smaller than the mean $\bar{\Phi_{b0}}$ unless $\frac{\sigma_{S}^{2}}{kT}\approx 0$. This is because TE occurs through lower barriers. From Equation (6), a plot of Φ_b0_ versus 1/2 kT is a straight line with the slope (*σ*_S_) and intercept ($\bar{\Phi_{b0}}$). Figure 4 shows the plots for Φ_b0_ versus 1/2 kT for different gate biases in the temperature range of 130–310 K where two straight lines with different slopes and intercepts are seen in the temperature range of 310–190 K (distribution 1) and 190–130 K (distribution 2). The values of $\bar{\Phi_{b0}}$ and *σ*_S_ are 0.65 eV and 119 meV for 2 V, 0.62 eV and 118 meV for 3 V, 0.61 eV and 117 meV for 4 V, and 0.59 eV and 116 meV for 5 V in the temperature range of 310–190 K. In the temperature range of 190–130 K, the values of $\bar{\Phi_{b0}}$ and *σ*_S_ come out to, respectively, be 0.45 eV and 84 meV for 2 V, 0.43 eV and 81 meV for 3 V, 0.41 eV and 80 meV for 4 V, and 0.39 eV and 79 meV for 5 V.

Table 1 shows that the lower mean barrier heights, $\bar{\Phi_{b0}}$ in the low-temperature region (190–130 K, distribution 2) are due to the surface-related traps [14], and the higher values of the mean $\bar{\Phi_{b0}}$ in the high-temperature region (310–190 K, distribution 1) are due to temperature-assisted tunneling [14]. In addition, the lower value of *σ*_S_ in the 190–130 K temperature range suggests more GaN nanowire homogeneity in this range compared to the 310–190 K range. There are several reports on double Gaussian distributions in GaN-based devices that can be ascribed to the nature of the inhomogeneities in the two regions [29,30]. These two regions of inhomogeneity may be related to variation in the interface phase/composition, electrical charges, interface quality, or nonstoichiometry, etc. In addition, such inhomogeneities may happen on a nanoscale that inhibits their detection using typical measurements. The inhomogeneities affect the *I*_ds_–*V*_gs_ measurements of a device mostly at low temperatures, so these measurements can explore the role of the barrier inhomogeneities present in the device. The occurrence of a double Gaussian distribution at a low temperature might happen due to some phase changes taking place below a certain temperature [26,31,32]. Further, the range of temperatures covered by each straight line suggests a region where the corresponding distribution is effective. The above results reveal that the temperature-dependent characteristics of the GaN nanowire WGT measured at the AlGaN/GaN interface in the source/drain regions can be explained by the presence of a double Gaussian distribution of the barrier heights.

## 4. Conclusions

In summary, the barrier height, ideality factor and role of inhomogeneities in GaN nanowire WGTs were studied at different gate potentials as a function of temperature. In I temperature range of 130–310 K, the experimental zero bias depletion width is nearly equal to the nanowire height. Therefore, we suggest that electrical transport through the nanowire is also affected by a tunneling mechanism. Φ_b0_ seems to decrease and η seems to increase with a decrease in temperature for all gate voltages. We ascribe these behaviors to barrier inhomogeneities in the GaN nanowires. The temperature-dependent *I*_ds_–*V*_gs_ characteristics of the GaN nanowire WGTs were shown to be a double Gaussian distribution with different standard deviations and mean barrier heights within the temperature regions of 310–190 K (distribution 1) and 190–130 K (distribution 2). These results are significant for the advancement of future applications and the enhancement of device performance.

## Figures and Tables

**Table 1:** Mean values of barrier heights and standard deviation at distribution 1 and 2 for different gate voltages.

*V*_gs_ (V)	Distribution 1		Distribution 2	
	$\bar{\Phi_{b0}}$ (eV)	*σ*_S_ (meV)	$\bar{\Phi_{b0}}$	*σ*_S_ (meV)
2	0.65	119	0.45	84
3	0.62	118	0.43	81
4	0.61	117	0.41	80
5	0.59	116	0.39	79

## Data Availability

The data are available on reasonable request from the corresponding author.

## References

[1] Lieber C.M., Wang Z.L. (2007). Functional nanowires. MRS Bull..

[2] Yang P., Yan R., Fardy M. (2010). Semiconductor nanowire: What’s next?. Nano. Lett..

[3] Calarco R., Stoica T., Brandt O., Geelhaar L. (2007). Surface-induced effects in GaN nanowires. J. Mater. Res..

[4] Im K.-S. (2023). Impact of fin width on low-frequency noise in AlGaN/GaN finFETs: Evidence for bulk conduction. IEEE Access.

[5] Doyle B.S., Boyanov B.S., Datta S.M., Doczy M.L., Hareland S., Jin B., Kavalieros J.T., Linton T.M., Rios R., Chau R. Tri-gate fully-depleted CMOS transistors: Fabrication, design and layout. Proceedings of the 2003 Symposium on VLSI Technology. Digest of Technical Papers (IEEE Cat. No. 03CH37407).

[6] Im K.-S., Sindhuri V., Jo Y.-W., Son D.-H., Lee J.-H., Cristoloveanu S. (2015). Fabrication of AlGaN/GaN Ω-shaped nanowire fin-shaped FETs by a top-down approach. Appl. Phys. Express.

[7] Im K.-S., Reddy M.S.P., Caulmione R., Theodorou C.G., Ghibaudo G., Cristoloveanu S., Lee J.-H. (2019). Low-frequency noise characteristics of GaN nanowire gate-all-around transistors with/without 2-DEG channel. IEEE Trans. Electron Devices.

[8] Im K.-S., An S.J., Theodorou C.G., Ghibaudo G., Cristoloveanu S., Lee J.-H. (2020). Effect of gate structure on the trapping behavior of GaN junctionless finFETs. IEEE Electron Device Lett..

[9] Singh N., Lim F.Y., Wang W.W., Rustagi S.C., Bera L.K., Agarwal A., Tung C.H., Hoe K.M., Omampuliyur S., Tripathi D. Ultra-narrow silicon gate-all-around CMOS devices: Impact of diameter, channel-orientation and low temperature on device performance. Proceedings of the 2006 International Electron Devices Meeting.

[10] Mallem S.P.R., Puneetha P., Choi Y., Baek S.M., An S.J., Im K.-S. (2023). Temperature-dependent carrier transport in GaN nanowire wrap-gate transistor. Nanomaterials.

[11] Mallem S.P.R., Puneetha P., Lee D.-Y., Kim Y., Kim H.-J., Im K.-S., An S.J. (2023). Carrier trap and their effects on the surface and core of AlGaN/GaN nanowire wrap-gate transistor. Nanomaterials.

[12] Konar A., Mathew J., Nayak K., Bajaj M., Pandey R.K., Dhara S., Murali K.V.R.M., Deshmukh M.M. (2015). Carrier transport in high mobility InAs nanowire junction-less transistors. Nano Lett..

[13] Moon B.H., Jan G.H., Kim H., Choi H., Bae J.J., Kim J., Jin Y., Jeong H.Y., Joo M.K., Lee Y.H. (2017). Junction-structure-dependent Schottky barrier inhomogeneity and device ideality of monolayer MoS_2_ field-effect transistors. ACS Appl. Mater. Interfaces.

[14] Dyakonova N., Dickens A., Shur M.S., Gaska R., Yang J.W. (1998). Temperature dependence of impact ionization in AlGaN-GaN heterostructure field effect transistors. Appl. Phys. Lett..

[15] Arulkumaran S., Egawa T., Ishikawa H., Jimbo T. (2003). Temperature dependence of gate-leakage current in AlGaN/GaN high-electron-mobility transistors. Appl. Phys. Lett..

[16] Sze S.M., Ng K.K. (2007). Physics of Semiconductor Devices.

[17] Yu L.S., Qiao D.J., Xing Q.J., Lau S.S., Boutros K.S., Redwing J.M. (1998). Ni and Ti Schottky barriers on n-AlGaN grown on SiC substrates. Appl. Phys. Lett..

[18] Ahmetoglu M., Akay S.K. (2010). Determination of the parameters for the back-to-back switched Schottky barrier structures. Curr. Appl. Phys..

[19] Zhang Z.Y., Yao K., Liu Y., Jin C.H., Liang X.L., Chen Q., Peng L.M. (2007). Quantitative analysis of current-voltage characteristics of semiconducting nanowires: Decoupling of contact effects. Adv. Funct. Mater..

[20] Kim J.-R., Oh H., So H.M., Kim J.-J., Kim J., Lee C.J., Lyu S.C. (2002). Schottky diodes based on a single GaN nanowire. Nanotechnology.

[21] Kolkovsky V., Sytkiewicz Z.R., Sobanska M., Klosek K. (2013). Electrical characterization of ensemble of GaN nanowires grown by the molecular beam epitaxy technique. Appl. Phys. Lett..

[22] Motayed A., Davydov A.V., Vaudin M.D., Levin I. (2006). Fabrication of GaN-based nanoscale device structures utilizing focused ion beam induced Pt deposition. J. Appl. Phys..

[23] Lee S.Y., Lee S.K. (2007). Current transport mechanism in a metal-GaN nanowire Schottky diode. Nanotechnology.

[24] Smit G.D.J., Rogge S., Klapwijk T.M. (2002). Enhanced tunneling across nanometer-scale metal-semiconductor interfaces. Appl. Phys. Lett..

[25] Kumar A., Heilmann M., Latzel M., Kapoor R., Sharma I., Gobelt M., Christiansen S.H., Kumar V., Singh R. (2016). Barrier inhomogeneities limited current and 1/*f* noise transport in GaN based nanoscale Schottky barrier diodes. Sci. Rep..

[26] Reddy M.S.P., Kumar A.A., Reddy V.R. (2011). Electrical transport characteristics of Ni/Pd/n-GaN Schottky barrier diodes as a function of temperature. Thin Solid Films.

[27] Werner J.H., Guttler H.H. (1991). Barrier inhomogeneities at Schottky contacts. J. Appl. Phys..

[28] Guttler H.H., Werner J.H. (1990). Influence of barrier inhomogeneities on noise at Schottky contacts. Appl. Phys. Lett..

[29] Kumar A., Nagarajan S., Sopanen M., Kumar V., Sing R. (2015). Temperature dependent 1/*f* noise characteristics of the Fe/GaN ferromagnetic Schottky barrier diode. Semicond. Sci. Technol..

[30] Kumar A., Kashid R., Ghosh A., Kumar V., Sing R. (2016). Enhanced thermionic emission and low 1/*f* noise in exfoliated graphene/GaN Schottky barrier diode. ACS Appl. Mater. Interfaces.

[31] Chand S., Kumar J. (1999). Evidence for the double distribution of barrier heights in Pd_2_Si/n-Si Schottky diodes from *I-V-T* measurements. Semicond. Sci. Technol..

[32] Vanalme G.M., Goubert L., Van Meirhaeghe R.L., Cardon F., Van Daele P. (1999). A ballistic electron emission microscopy study of barrier height inhomogeneities introduced in Au/III-V semiconductor Schottky barrier contacts by chemical pretreatments. Semicond. Sci. Technol..

